# Co-circulation and co-infection of hantaviruses and Wenzhou mammarenavirus in small mammals and humans in Jiangxi, China

**DOI:** 10.3389/fmicb.2023.1225255

**Published:** 2023-07-12

**Authors:** Shanshan Du, Yun Xie, Xuefei Deng, Zhiyong Xia, Wei Wu, Xiaoxia Huang, Zhao Chen, Aqian Li, Chuan Li, Qin Wang, Lina Sun, Meijun Guo, Shiwen Wang, Mifang Liang, Dexin Li, Xiaoqing Liu, Jiandong Li

**Affiliations:** ^1^NHC Key Laboratory of Biosafety, NHC Key Laboratory of Medical Virology and Viral Diseases, National Institute for Viral Disease Control and Prevention, China CDC, Beijing, China; ^2^Jiangxi Province CDC, Nanchang, Jiangxi, China; ^3^Gao’an City CDC, Gao’an, Jiangxi, China; ^4^Yichun City CDC, Yichun, Jiangxi, China

**Keywords:** hantavirus, Wenzhou mammarenavirus, rodents, co-infection, HFRS, Seoul virus (SEOV), Hantaan virus (HTNV)

## Abstract

Both Orthohantaviruses (HV) and Whenzhou Mammarenaviruses (WENV) are rodents borne viruses, allowing them to spread simultaneously in the same area and infect humans. To explore the potential threat of HV and WENV to public health safety, an environmental and laboratory investigation was conducted in 2020–2021, in Jiangxi province, China. A total of 461 small mammals of 7 species and paired sera from 43 suspected HFRS cases were collected from Jiangxi Province, China. Viral genomic RNA and specific antibodies against HV and WENV were detected to evaluate the epidemic situation of the two viruses. Hantaan virus (HTNV), seoul virus (SEOV) and WENV RNA were detected in the lungs of the captured mammals, which resulted 4.1% and 7.4% of HV and WENV RNA positive respectively. Co-infections of WENV and SEOV were detected from Rattus norvegicus, Mus musculus and Rattus flavipectus with an overall co-infection rate of 0.65%. The detection rates of antibodies in the blood against HV and WENV were 11.9% (55/461), and 13.2% (61/461) respectively. The prevalence of viral infection and viral genetic characters varied among the selected areas. In the paired sera of 43 suspected HFRS cases, 38 were with HV infection, 11 were with WENV IgG, and 7 with a 4-fold or more of WENV IgG titer elevation. These results revealed the fact of the co-circulating and coinfection of HV and WENV in the same area at the same time, which might impact on public health safety.

## Introduction

Emerging infectious diseases are a great threat to global public health (Jones et al., 2008). Zoonoses account for a large percentage of new and existing human diseases (Woolhouse and Gowtage-Sequeria, 2005). As the most abundant and widely distributed mammals, rodents are reservoirs for a number of zoonotic viral pathogens with public health significance (Mills, 2006). Rodent-borne *orthohantaviruses* and *mammarenaviruses* can generally cause chronic or persistent infections in rodents and are continuously excreted in urine and saliva for weeks or even months, leading to hundreds of thousands of human infections each year (Schmaljohn and Hjelle, 1997; Charrel and de Lamballerie, 2010).

Wenzhou virus (WENV), which belongs to the *mammarenaviruses*, *Arenaviridae,* was first identified in rodents and Asian house shrews in Wenzhou, China, in 2014 and named after its place of discovery (Li et al., 2015). Subsequently, it was found to be widely distributed in China (Li et al., 2020; Wu et al., 2021). A serologic survey in the general population of 830 people aged from 1 to 70 years old showed that the seroprevalence of WENV IgG antibody was 4.6% (Li et al., 2020). In 2015, WENV genomic RNA was detected in the respiratory tract samples of rodents collected from Cambodia, Thailand, and Laos, and WENV-specific IgG antibodies were detected by ELISA with a positive rate of 17.4% (89/510) in the sera collected from healthy individuals and patients with dengue/influenza-like illness (Blasdell et al., 2016; Artois et al., 2018). Hantaviruses (HV) are a family of viruses that can cause various disease syndromes in humans worldwide (Schmaljohn and Hjelle, 1997). Hantaviruses in the Americas may cause hantavirus pulmonary syndrome (HPS), and those most commonly found in Europe and Asia may cause hemorrhagic fever with renal syndrome (HFRS) (Schmaljohn and Hjelle, 1997). HFRS has been reported in China for decades and still poses a significant threat to human health (Wei et al., 2021). Hantaan virus (HTNV) and Seoul virus (SEOV) are the main causes of HFRS in China (Schmaljohn and Hjelle, 1997; Wei et al., 2021). Jiangxi Province is typically a natural focus of HFRS, with surveillance sites for monitoring hantavirus infection in humans and rodents having been established since the 1980s. Since then, there have been a small number of suspected cases of HFRS that could not be confirmed or ruled out by the laboratory tests reported annually. Most of these cases were self-limited diseases with mild clinical manifestations and did not pose a significant public health threat. As the epidemic characteristics of the WENV were gradually elucidated, it was suspected that it might be related to some of these HFRS-like cases. To explore the possible causes of these suspected cases and to evaluate the epidemic situation and the potential threat of HV and WENV to public health safety, an environmental and laboratory investigation was conducted at the HFRS surveillance sites in Jiangxi Province in 2020–2021.

## Materials and methods

### Sample collection

Small mammals were sampled at selected residential, farmland, and forest localities in Gao An (GA), Tong Gu (TG), and An Yi (AY) in Jiangxi Province, China, in 2020–2021 (Appendix Figure S1). At each site, small mammals were captured in traps baited with a mixture of peanut and maize flour. If trapping success was low after two nights, additional sites were selected at a minimum distance of 500 m and a maximum distance of 2 km. The captured animals were collected early in the morning and transported to the local CDC laboratories in a box with ice bags for species identification, dissection, and specimen collection. Blood was drawn from the punctured heart with a capillary tube and preserved on pre-punched filter paper, and kept at 4°C for a maximum of 3 days for detection prior to storage at −80°C, and organ samples were preserved at −80°C or directly in liquid nitrogen.

The hospitalized patients who had been clinically diagnosed with HFRS in the counties of GA, TG, and AY in 2020–2021 were selected for evaluation of HV and WENV infections. Serum samples were collected during the acute phase, preferably within 2 weeks after the onset of fever, and during the convalescent phase (within 15–45 days after the onset of illness).

### ELISA

Antibodies to WENV and HV in the sera of captured small mammals were detected using a double antigen sandwich ELISA as previously described (Niu et al., 2013). Briefly, a His-tagged nucleocapsid (N) protein purified by affinity chromatography of WENV (GenBank accession NC026018.1), HTNV (strain 84Fli), and SEOV (strain L99)was prepared in *Escherichia coli* BL21 (DE3) and used as the coating antigen for 96-well ELISA plates, and horseradish peroxidase (HRP)-conjugated N protein was used for detection. Dried blood spots on the filter papers were soaked in 500 μL of extraction buffer phosphate-buffered saline (PBS). Serums obtained from N protein-immunized and non-immunized laboratory *Rattus norvegicus* were used as positive and negative controls, respectively. All mammalian blood samples, including controls, were tested at a dilution of 1:10. After coating the ELISA plates with purified N protein (0.4 μg/well), 100 μL of diluted blood samples were added per well, followed by HRP-conjugated N protein.

To detect WENV-specific IgG antibodies in the sera of HFRS patients in both the acute and convalescent stages, an indirect IgG ELISA was used. Briefly, the plate was coated with purified N protein as described, and two-old dilutions of serum samples were added, followed by adding HRP-conjugated anti-human IgG antibody (Sigma, Saint Louis, United States). HV-specific IgG and IgM in patient sera were detected using an indirect IgG ELISA and MacELISA method with commercial HFRS IgG and IgM detection kits, respectively, according to the manufacturer’s instructions (Wangtai, China). Endpoint titers of IgG to WENV and HV were expressed as the reciprocal of the highest dilution of the sera.

TMB peroxidase substrate [3,3′,5,5-tetramethylbenzidine and hydrogen peroxide (H_2_O_2_)] was used for color development, and substrate conversion was measured using a DTX 880 multimode detector (Beckman Coulter, CA, United States) with an incidence wavelength of 450 nm and a reference wavelength of 620 nm. Cut-off values were determined as the mean absorbance of the negative control wells multiplied by a factor of 2.1. A sample was considered positive if the OD value was above the cut-off threshold.

### TaqMan quantitative real-time RT-PCR assay

The TaqMan quantitative real-time RT-PCR (qRT-PCR) assay was performed on all lung tissue samples from field animals and human sera samples. The genomic sequences of WENV were collected from the GenBank database, and specific primers and probes were designed after alignment with software (Appendix Table S1). The previously described primers and probes were used for the detection of HTNV and SEOV (Pang et al., 2014). The amplification plots and standard curves of these one-step real-time TaqMan RT-PCR assays are shown in (Appendix Figure S2). The detection limits for HTNV, SEOV, and WENV in tissue samples were 150, 50, and 70 copies per reaction, respectively. The reference RNA for these detection methods was obtained by an *in vitro* transcription method using RiboMAX^™^ Large Scale RNA Production Systems-SP6 and T7 Kit (Promega, China) with related chemically synthesized target genes as templates. All the oligos and DNA were synthesized by Sangong Bioengineering Co., Ltd. (Sangong, Shanghai, China). The QiaAmp viral RNA Mini Kit was used for RNA extraction of virus samples, simulated synthetic RNA samples, and human serum samples; the RNeasy Mini Kit was used for RNA extraction from tissues and cells according to the kit instructions. A one-step fluorescent quantitative RT-PCR kit (AgPath-ID Onestep RT-PCR Kit, ABI) was used for viral RNA detection. The reaction conditions were 50°C for 30 min; 95°C for 10 min, 95°C for 15 s, 60°C for 45 s, 40 cycles. Viral RNA copy numbers were determined by amplification of a standard curve of positive control RNA, and the cut-off cycle threshold (Ct) value for a positive reaction was set at 35 cycles.

### Sequence analysis and phylogenetic analyses

The tested positive samples and isolated viruses were amplified by degenerate primers and sequenced using Sanger sequencing. The resulting viral genomic sequences and open reading frame (ORF) amino acid sequences were aligned with arenavirus and hantavirus sequences available in GenBank. All sequence alignments for phylogenetic analysis were performed using Clustal W embedded in Mega11 software. Sequence lengths were trimmed to the same length when including representative sequences available in the public database, and the phylogenetic tree was constructed by neighbor-joining (NJ) using Mega11 (Tamura et al., 2021).

### Statistical analyses

The rates of viral RNA and antibody detection were calculated by species, and statistical significance was analyzed using either the Pearson *χ*^2^ test or the continuity correction *χ*^2^ test. Analyses were performed using the SPSS software (ver. 16.0). Values of *p* < 0.05 were considered statistically significant.

### Ethics statement

All animal work and human sample collection were reviewed and approved by the Ethics Committee of the National Institute for Viral Disease Control and Prevention, China CDC, according to the medical research regulations of the Ministry of Health, China.

## Results

### Prevalence and co-infections in small mammals

A total of 461 small mammals were captured from the selected fields and residential areas in GA, TG, and AY, Jiangxi Province (Table 1). Among them, 99.15% of the 235 *Apodemus agrarius*, 60.87% of the 23 *Rattus losea,* 46.15% of the 13 *Rattus confucianus*, and 58.33% of the 24 *Suncus murinus* were caught in the fields, and the rest were caught in residential localities. In addition, 82 *R. norvegicus*, 63 *Rattus flavipectus*, and 21 *M. musculus* were collected from residential areas. Lung and blood samples were collected from each animal and tested with a total HV antibody detection rate of 11.9%, a HV RNA detection rate of 4.1%, a WENV antibody detection rate of 13.2%, and a WENV RNA detection rate of 7.4% (Table 1).

**Table 1:** Summary of the detection of viral RNA and specific antibodies in the captured small mammals, in Jiangxi Province in 2020–2021.

Region	Habitat	Animal host	Number of animals captured	Number of hantavirus-Ab positive (%)	Number of hantavirus RNA positive (%)	Number of WENV-Ab positive (%)	Number of WENV-RNA positive (%)
Gao An	Field	*A. agrarius*	104	15 (14.4)	2 (1.9)	16 (15.4)	10 (9.6)
		*R. confucianus*	3	0	0	0	1 (33.3)
		*R. losea*	9	2 (22.2)	1 (11.1)	4 (44.4)	2 (22.2)
		*S. murinus*	2	0	0	1 (50)	1 (50)
	Residential area	*R. confucianus*	1	0	0	0	0
		*R. losea*	9	5 (55.6)	1 (11.1)	0	0
		*S. murinus*	0	0	0	0	0
		*R. flavipectus*	19	1 (5.3)	0	5 (26.3)	5 (26.3)
		*R. norvegicus*	54	2 (3.7)	5 (9.3)	12 (22.2)	11 (20.4)
		*M. musculus*	7	0	0	1 (14.3)	1 (14.3)
Total			208	25 (12.0)	9 (4.3)	39 (18.8)	30 (14.4)
An Yi	Field	*A. agrarius*	86	16 (18.6)	6 (7.0)	4 (4.7)	2 (2.3)
		*R. confucianus*	3	0	0	0	0
		*R. losea*	5	0	0	2 (40)	0
		*S. murinus*	6	2 (33.3)	0	1 (16.7)	0
	Residential area	*A. agrarius*	2	1 (50)	0	1 (50)	0
		*R. confucianus*	6	1 (16.7)	0	1 (16.7)	0
		*S. murinus*	10	1 (10)	0	3 (30)	0
		*R. flavipectus*	44	4 (9.1)	2 (4.5)	2 (4.5)	0
		*R. norvegicus*	28	2 (7.1)	1 (3.6)	2 (7.1)	1 (3.6)
		*M. musculus*	14	2 (14.3)	0	5 (35.7)	1 (7.1)
Total			204	29 (14.2)	9 (4.4)	21 (10.3)	4 (1.96)
Tong Gu	Field	*A. agrarius*	43	1 (2.3)	1 (2.3)	0	0
		*S. murinus*	6	0	0	1 (16.7)	0
	Residential area		0	0	0	0	0
Total			49	1 (2)	1 (2)	1 (2)	0
Total			461	55 (11.9)	19 (4.1)	61 (13.2)	34 (7.4)

**Figure 1:**
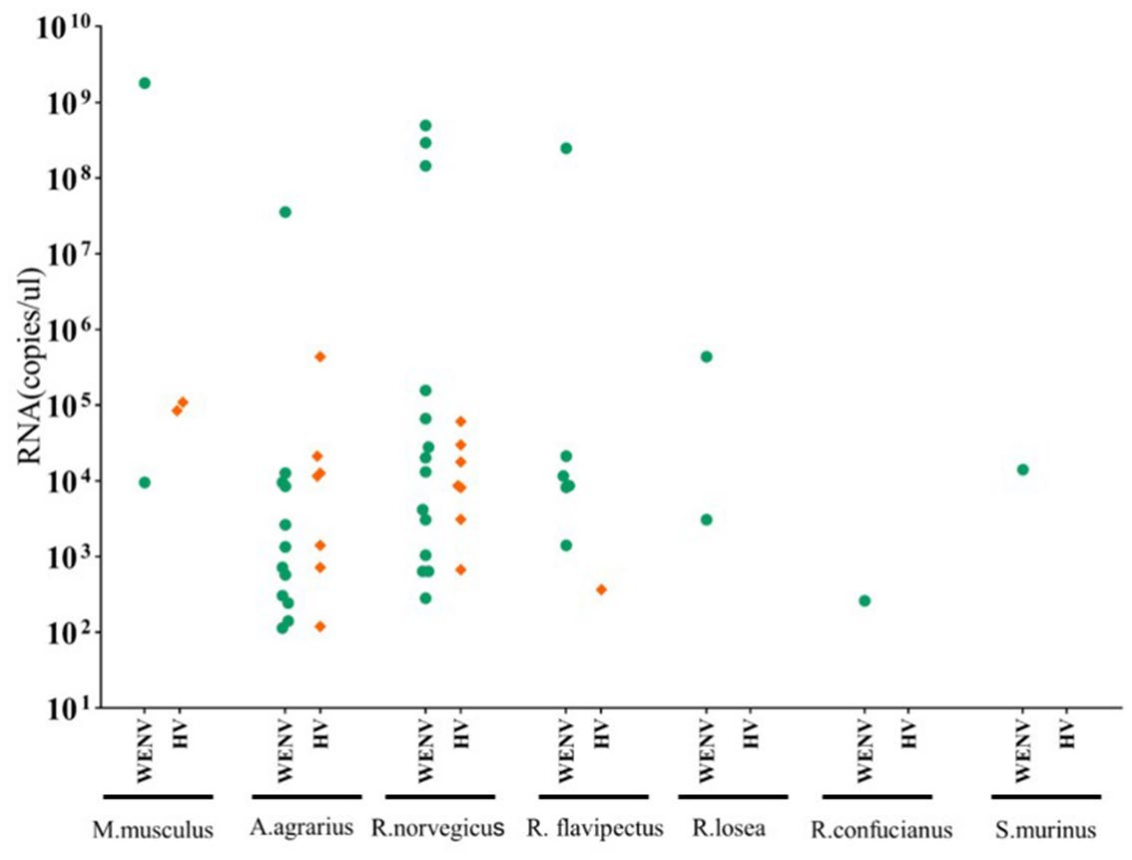
Viral RNA copies in lung tissue samples from captured small mammals. Viral RNA copies in the captured small mammals of seven species were quantified using real-time RT-PCR, and copy numbers in animals positive for viral RNA are shown. In the scatter dot graphs, each dot represents a sample, and copy numbers are indicated as green circular dots (HV) and orange diamonds (WENV), respectively. The detection limits for HTNV, SEOV, and WENV in tissue samples were 150, 50, and 70 copies per reaction, respectively.

Specific antibodies to WENV and HV, in addition to genomic RNA, were detected in the seven captured small mammal species, and although the detection rates varied, all seven small mammal species were shown to be infected with HV and WENV. HTNV was detected in the lung samples of *A. agrarius* at a rate of 3.83% (9/235), and the detection rate in AY (6.98%, 6/86) was significantly higher than that in GA (1.92%, 2/104) and TG (2.32%, 1/43). SEOV were detected in the other species of small mammals captured; *R. norvegicus* captured in GA showed a detection rate (9.3%, 5/54) higher than that in AY and TG. The overall positive viral RNA detection rates for WENV were higher than in HV and varied among the three counties, with the variance being significant (*χ*^2^ = 27.78, *p* < 0.01). In GA, the detection rate of WENV RNA in *R. norvegicus* (20.4%), *R. flavipectus* (26.3%), *M. musculus* (14.3%), and *A. agrarius* (9.6%), was higher than that in AY and TG, respectively (*χ*^2^ = 29.94, *p* < 0.01). Both WENV and SEOV genomic RNA were detected from one *R. norvegicus*, one *M. musculus,* and one *R. flavipectus* captured from GA, with an overall co-infection rate of 0.65%. The detected RNA concentration of WENV in the lungs of the captured small mammals was significantly higher than that of HV (*χ*^2^ = 4.50, *p* = 0.03). The detected HV RNA was in the range of 10^3^ to 10^5^ copies/μL, with a Ct value of 24 to 31 when detected by qRT-PCR assay. However, the concentration of WENV RNA could be as high as 10^9^ copies/μL (Figure 1).

The detection rates of WENV antibodies in *R. norvegicus, A. agrarius*, and *R. flavipectus* captured in GA were significantly higher than those in AY and TG (*χ*^2^ = 24.38, *p* < 0.01, Table 1). Antibodies to HV and WENV were detected with an overall co-infection rate of 1.74% in three *A. agrarius* (2.88%, 3/104), two *R. flavipectus*, one *R. norvegicus,* one *S. murinus,* and one *M. musculus*, respectively.

### Prevalence and co-infections in humans

To investigate the virus infection in humans, paired serum samples collected from 43 clinically diagnosed HFRS patients during the acute and convalescent phases were tested for seroconversion of IgG antibodies (Table 2). Antibodies to hantavirus were detected in 38 of the 43 patients with IgM positivity, a four-fold increase in IgG titer, or IgG antibody seroconversion (Table 2). The increase in antibody titers in the paired serum samples was expected, as these serum samples were collected from patients in the acute and convalescent phases, respectively. Of the other five patients, one (SLL43) was IgG positive without an increase in antibody titer in the paired samples, and four showed no detectable hantavirus-specific antibodies, suggesting that the diagnosis of HFRS could be excluded. WENV-specific IgG was detected in the convalescent serum of 11 patients (25.58%, 11/43), and IgG appeared in the serum collected during the acute phase from four patients. A four-fold increase in WENV IgG antibody titer and/or seroconversion was found in seven patients (Table 2), six of whom were confirmed to be HFRS cases by positive IgM detection or elevated hantavirus IgG antibody titers, while a titer of more than 1,600 of antibody against WENV in ELISA was found in three convalescent sera, suggesting a strong humoral immune response against WENV. In addition, one male patient (LNF39) was found to have only a 4-fold increase in IgG titer to WENV in the paired serum samples, a test result that usually meets the criteria for laboratory confirmation. However, no WENV RNA was detected in serum collected during the acute phase from 43 patients.

**Table 2:** IgG and IgM antibody detection in paired serum samples from suspected HFRS patients.

Patients	Gender	Age	Occupation	Days (AP)^a^	Days (CP)^b^	WENV-IgG^e^		HFRS IgG^e^		HFRS-IgM
						AP^c^	CP^d^	AP	CP	AP
LYY01	F	59	Farmer	3	21	0	0	200	400	+
XGY04	M	9	Student	3	21	0	0	800	800	+
WCC05	M	14	Student	5	18	0	0	400	400	+
FCY06	F	54	Farmer	3	17	0	0	0	0	−
WH08	M	10	Student	2	21	0	0	200	800	+
HZH11	M	29	Farmer	4	20	0	0	200	800	+
LQX12	M	3	Child	5	18	0	0	200	400	+
CSL13	M	70	Farmer	5	25	0	0	100	400	+
FSY16	F	37	Farmer	5	28	0	0	100	800	+
LXZ20	F	79	Farmer	3	25	0	0	800	800	+
AJG21	M	72	Farmer	2	18	0	0	0	800	+
LXL22	M	20	Student	5	18	0	0	800	800	+
XYC23	M	18	Student	6	19	0	0	800	800	+
GSH24	M	66	Retired	5	16	0	0	100	400	+
XZY25	M	58	Farmer	4	21	0	0	100	400	+
HSC26	M	9	Student	4	20	0	0	100	800	+
ZJM27	F	52	Farmer	3	21	0	0	100	400	+
HYR32	F	71	Farmer	4	20	0	0	0	800	+
XF33	F	8	Student	6	28	0	0	200	800	+
ZXR34	M	57	Houseworker	5	24	0	0	400	1,600	+
YXF36	F	34	Farmer	5	23	0	0	400	800	+
WF37	M	33	Farmer	5	23	0	0	100	800	+
WTZ38	M	63	Retired	6	21	0	0	100	800	+
TSX40	M	79	Farmer	5	23	0	0	200	800	+
LYJ41	M	15	Student	4	20	0	0	200	200	+
HLM42	F	68	Farmer	5	21	0	0	200	400	+
SLL43	F	55	Farmer	5	26	0	0	400	400	−
HFA44	M	71	Farmer	7	24	0	0	0	0	−
ZSJ45	M	64	Farmer	3	34	0	0	0	400	+
TJQ46	M	75	Farmer	6	22	0	0	0	0	−
LAM47	F	60	Farmer	3	18	0	0	100	400	+
WXH49	M	44	Officer	4	18	0	0	200	800	+
LDY28	M	78	Farmer	4	20	0	100	0	400	+
XL02	F	19	Farmer	5	20	0	400	100	400	+
WJQ48	M	57	Farmer	7	25	0	400	0	400	+
LNF39	M	50	Farmer	4	31	0	800	0	0	−
LHX29	F	54	Farmer	5	23	0	>1,600	100	100	+
LZX18	F	25	Houseworker	5	17	100	>1,600	100	800	+
LKH31	M	13	Student	4	22	100	>1,600	100	100	+
MLY07	F	29	Farmer	5	21	400	400	400	400	+
WYY10	F	65	Farmer	2	27	400	400	400	400	+
ZEY14	F	34	Farmer	6	17	400	400	400	400	+
WPJ15	M	60	Farmer	5	22	400	400	100	400	+

a Number of days after onset when acute phase (AP) samples were collected.

b Number of days after onset when samples were collected in the convalescent phase (CP).

c Patient samples collected in the acute phase.

d Patient samples collected in the convalescent phase.

e The value expressed is the reciprocal of the dilution ratio.

### Phylogenetic analysis and identity comparisons

**Figure 2:**
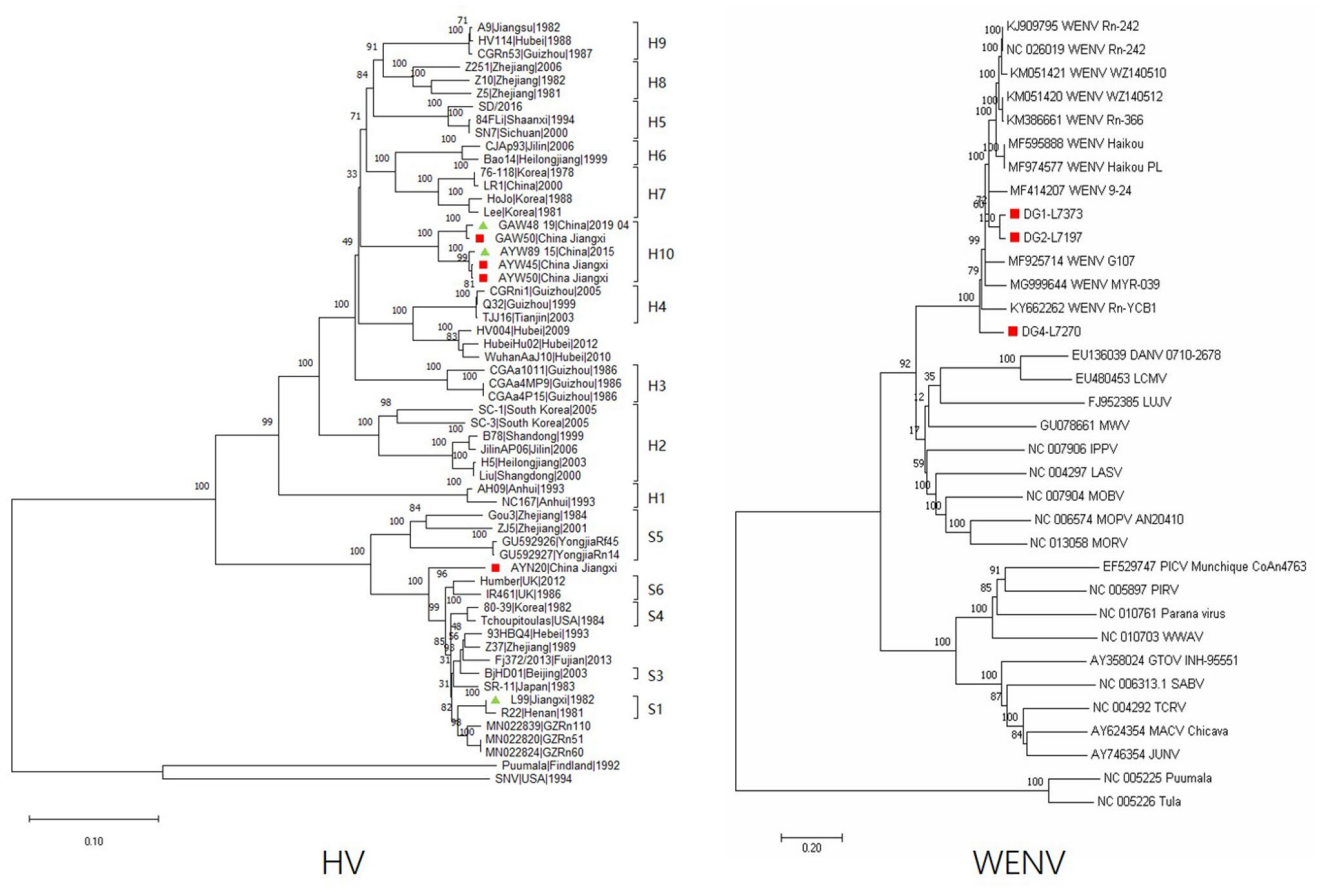
Phylogeny of the partial M segment (1843 nucleotides) of HV and the complete L segment (7,197 nucleotides) of WENV amplified from the lung tissue of the captured rodents. Sequences indicated in the red cubic block represent viral sequences detected in this study; the green triangle indicates that the corresponding sequence was previously obtained in the study area but not in this study. The evolutionary history was inferred using the neighbor-joining method. The optimal tree is shown, and the percentage of replicate trees in which the associated taxa clustered together in the bootstrap test (1,000 replicates) is shown above the branches. Evolutionary distances were computed using the Maximum Composite Likelihood method and are expressed in terms of the number of base substitutions per site. Evolutionary analyses were conducted in MEGA11. The sequences of DG1-L7373 were obtained from *M. musculus*, DG2-L7197 were obtained from *R. norvegicus*, and DG4-L7270 were obtained from *R. flavipectus*; the sequences of GAW50, AYW50, AYW45, GAW48, and AYW89 were obtained from *A. agrarius*; and the sequence of AYN20 was obtained from *M. musculus.*

The complete nucleotide sequences of the L segments of WENV were obtained from the RNA-positive lung samples of *M. musculus* (DG1, 7,373 nt), *R. norvegicus* (DG2, 7,197 nt), and *R. flavipectus* (DG4 7,270 nt) by RT-PCR, respectively. The collected sequences were aligned with representatives of mammarenaviruses, and the selected genomic sequences of Puumala and Tula viruses were used as outgroups to construct phylogenetic trees (Figure 2). The results showed that the genomic sequences of DG1, DG2, and DG4 were clustered into the Wenzhou virus clade. The nucleotide similarity comparisons among the selected Wenzhou viruses were in the range of 81.1%–99.7%, and the sequences obtained in this study shared a nucleotide similarity with the selected strains in the range of 81.1%–92.3%. DG1 and DG2 showed a nucleotide similarity of 92.3% and were clustered together into one lineage; DG4, the nucleotide sequence obtained from *R. flavipectus,* formed an independent lineage and shared a nucleotide similarity of 81.4% and 81.9% with DG1 and DG2, respectively.

Partial genomic sequences of 1791 nt of the hantavirus M segment were obtained and aligned with representatives of orthohantavirus selected from GenBank. The results showed that both Hantaan virus and Seoul virus are circulating in the study areas. The sequences of GAW48 and GAW50 obtained from *A. agrarius* in GA shared a nucleotide similarity of 98.9% and were clustered into one lineage (Figure 1). The sequence of AYW50 and AYW45, obtained from *A. agrarius* at AY, were clustered into the same lineage together with AYW89, obtained in 2015. AYW50 and AYW45 shared a nucleotide similarity of 99.6% and had a nucleotide similarity of 98.9% with the sequence of AYW89. The nucleotide sequences obtained from GA and AY shared a similarity of 94.9% and formed an independent clade, or genotype. The same result was found in the blast analysis in GenBank. The sequence of AYN20, obtained from *M. musculus* in AY, showed a similarity of about 93.3% to the closest sequence among the existing published SEOV sequences in GenBank and a nucleotide similarity of 91.7% to a reference SEOV strain L99 isolated in 1982 in Jiangxi Province, forming an independent clade in the constructed phylogenetic tree.

## Discussion

Rodents are the most widely distributed mammals in the world, are closely related to humans, and are recognized as important natural reservoirs of many zoonotic pathogens (Han et al., 2015). HV can cause various disease syndromes in humans worldwide, which have coevolved with *Murinae* rodents in China for a long time and still pose a significant threat to human health (Wei et al., 2021). WENV is a species of *mammarenavirus*, a genus containing a variety of notorious pathogens that can cause serious diseases in humans. It has been reported that RNA viruses with segmented genomes and complete replication in the cytoplasm are more likely to cause cross-species transmission (Cleaveland et al., 2001; Pulliam and Dushoff, 2009). A potential risk of segmented RNA viruses is their ability to exchange genome segments during the co-infection of two or more viruses in a single host cell, thereby producing hybrid progeny. This is one of the key mechanisms underlying the evolution of viruses.

In this study, detection of viral RNA and antibodies was performed in 461 captured small mammal and human samples from GA, AY, and TG of Jiangxi Province, resulting in 19 (4.1%) hantavirus RNA and 34 (7.4%) WENV RNA positives. Phylogenetic analysis of the obtained viral genome sequence showed that both HTNV and SEOV were circulating in the study areas. In hantaviruses, it is generally accepted that a new genotype of HV could be defined when the difference in viral genome sequence exceeds 5%. The sequence of HTNV obtained from *A. agrarius* in GA and AY, respectively, shared a nucleotide similarity of 94.9% and was clustered into an independent clade compared with the sequences obtained from other areas in China. The sequence of AYN20 showed 93.3% nucleotide similarity to the closest SEOV sequence among the existing published sequences in GenBank, suggesting a distinct genotype of the Seoul virus circulating in AY, Jiangxi Province. Three L-segment sequences of WENV were recovered from the lung samples of *M. musculus*, *R. norvegicus*, and *R. flavipectus*, respectively. The nucleotide similarity of the three sequences is in the range of 81.4%–92.3%, illustrating the extensive genetic diversity of WENV circulating in GA during the same period. The majority of the presently recognized viral species have been associated with a specific rodent species, but many of these have been shown to host multiple virus species (Mollentze and Streicker, 2020). One study reported that the recombination of WENV happened in nature based on a phylogenetic analysis of the S segment sequence (Wang et al., 2019). In this study, we detected WENV and SEOV RNA from *R. norvegicus*, *M. musculus,* and *R. flavipectus* captured from GA with an overall co-infection rate of 0.65%. It has been reported that the co-infection of different species of influenza viruses leads to the emergence of a reassortant virus with increased fitness for poultry and zoonotic potential (Bhat et al., 2022). The replication cycles of HV and WENV in the cytoplasm of the infected cells shared somewhat similar mechanisms; co-infection of the two viruses might increase the risk of potential adaptive recombination leading to emerging pathogenic variants. The rodent hosts of HV and WENV are widely distributed, with many of them, such as *R. norvegicus* and *M. musculus*, commonly found in human dwellings and peridomestic habitats, perhaps facilitating more frequent spillover of the viruses to humans. It is worthwhile to pay appropriate attention to the co-circulation and co-infection of HV and WENV in rodents and humans and consider including them in a syndromic monitoring system.

The detected RNA concentration of WENV in rodent lung samples was significantly higher than that of HV, which may suggest the risk of infected rodents excreting more infectious viruses, leading to a greater risk of WENV transmission and spread. This may be one of the factors leading to a significantly higher seroprevalence rate of WENV antibodies in GA compared to HV. In previous studies, WENV infections were detected in patients with dengue fever and influenza-like illnesses (Blasdell et al., 2016; Artois et al., 2018), but the pathogenic characteristics were still unclear. We collected paired serum samples from 43 suspected HFRS patients to evaluate the situation of HV and WENV infection; 38 were confirmed to have HV infection, four showed no detected hantavirus-specific antibodies, and one (SLL43) showed positive IgG detection without antibody titer elevation from the acute to the convalescent phase, which suggested that other pathogenic factors may exist in these patients with HFRS-like diseases. WENV-specific IgG was detected in 11 patients (25.58%, 11/43). A four-fold increase in WENV IgG antibody titer and/or seroconversion was found in seven patients, six of whom were confirmed with HV infection, while one male (LNF39) had a four-fold rise in IgG titer for WENV in the paired serum samples, which usually meets the criteria for laboratory confirmation. However, WENV RNA was not detected in the acute-phase serum sample. At the same time, as in a recent study (Tan et al., 2019), we did not obtain any infectious WENV by cell culture, which prevented a confirmatory evaluation of neutralizing antibodies in the paired serum samples. Therefore, whether WENV was the etiology of the case (LNF39) remains to be studied. However, the fact that WENV could induce a strong humoral immune response in humans suggests that WENV plays some role in human pathogenesis.

In conclusion, in this study, we presented molecular and serologic evidence of co-circulation and co-infection of HV and WENV among small mammals and humans in GA, AY, and TG, Jiangxi Province, China. Extensive genetic diversity of HV and WENV was shown by the detection of three clades of HV and two clades of WENV co-circulating in the study areas. Although further studies are still needed to elucidate the pathogenicity of WENV, the findings here showed the potential impact of WENV on human health and the potential risks of the emergence of virus variants facilitated by the co-circulation and co-infection of HV and WENV in the same period and in the same areas.

## Data availability statement

The datasets presented in this study can be found in online repositories. The names of the repository/repositories and accession number(s) can be found in the article/Supplementary material.

## Ethics statement

The studies involving human participants were reviewed and approved by The ethics committee of National Institute for Viral Disease Control and Prevention, China CDC. The patients/participants provided their written informed consent to participate in this study. The animal study was reviewed and approved by The ethics committee of National institute for Viral Disease Control and Prevention, China CDC.

## Author contributions

JL and XL contributed to the conception and design of the study. SD, YX, and XD collected the samples and performed detection and statistical analysis. ZX and ZC collected, processed, and transported the samples. WW, XH, AL, CL, QW LS, MG, SW, ML, and DL organized the database and laboratory detection. JL, SD, and XD wrote the draft of the manuscript. All authors contributed to the article and approved the submitted version.

## Funding

This work was supported by the Project of Capital Clinical Diagnosis and Treatment Technology Research and Transformation (Z221100007422076), and the Program for Infectious Disease Surveillance of China. The funders had no role in the study design, data collection and analysis, decision to publish, or preparation of the manuscript.

## Conflict of interest

The authors declare that the research was conducted in the absence of any commercial or financial relationships that could be construed as a potential conflict of interest.

## Publisher’s note

All claims expressed in this article are solely those of the authors and do not necessarily represent those of their affiliated organizations, or those of the publisher, the editors and the reviewers. Any product that may be evaluated in this article, or claim that may be made by its manufacturer, is not guaranteed or endorsed by the publisher.
