# Supplementary material for: Co-circulation and co-infection of hantaviruses and Wenzhou mammarenavirus in small mammals and humans in Jiangxi, China
Source: Front Microbiol. 2023 Jul 12;14:1225255. doi: 10.3389/fmicb.2023.1225255 (PMC10371193; doi:10.3389/fmicb.2023.1225255)
Supplement: Supplementary file 1 [file Data_Sheet_1.docx]

**Appendix Table 1. The sequence of primers and probes for detection of hantavirus and Wenzhou virus using real-time qRT-PCR assays**

| virus | Forward Primers | Reverse Primer | Probes | Amplicon size (bp) |
| --- | --- | --- | --- | --- |
| HTNV* | 5’GCTTCTTCCAGATACAGCAGCAG-3’ | 5’GCCTTTGACTCCTTTGTCTCCAT-3’ | 5’FAMCCTGCAACAAACAGGGAYTACTTACGGCA-BHQ1 | 114 |
| SEOV* | 5’GATGAACTGAAGCGCCAACTT-3’ | 5’CCCTGTAGGATCCCGGTCTT-3’ | 5’HEXCCGACAGGATTGCAGCAGGGAAGAA-BHQ1 | 76 |
| WENV | 5’ATYGTGCCGAACTGRTTGT-3’ | 5’AGCTGGCTCTGATGGTGTTG-3’ | 5’HEXTGTTCAGGAGATCTGGGTTCTTTACGTCCCAGAC-BHQ1 | 150 |

* The primers and probes were designed and evaluated by Pang Z，et al in reference:

Pang Z，Li AQ，Li JD，Qu J，He CC，Zhang S，Li C，Zhang QF，Liang MF，Li DX. Comprehensive Multiplex One‐Step Real‐Time TaqMan qRT‐PCR Assays for Detection and Quantification of Hemorrhagic Fever Viruses. PLoS One，2014，9（4）：e95635.

*
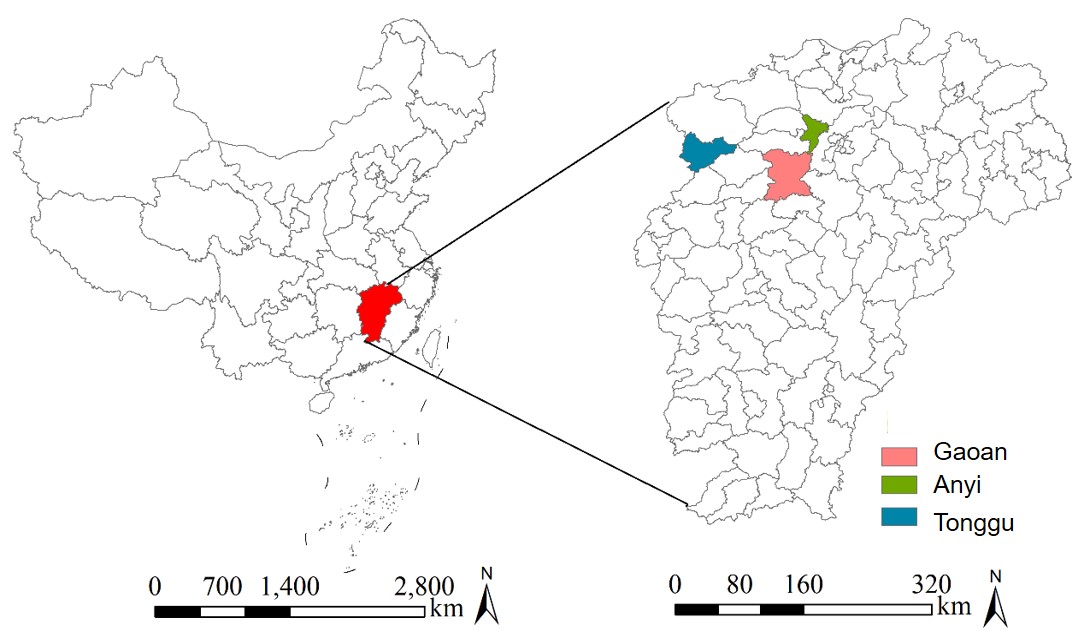
*

**Appendix Figure 1.** Location of Gaoan (GA), Anyi (AY) and Tonggu (TG) counties in Jiangxi province, China. Maps of GA, AY and TG Counties in Jiangxi Province, China, where small mammals were sampled and corresponding serum samples were collected in the study area in 2020-2021. Map at left side shows location of Jiangxi Province (red shading) in China; and map at right side shows GA (purple shading) and AY (green shading) and TG (blue shading) counties in Jiangxi Province.


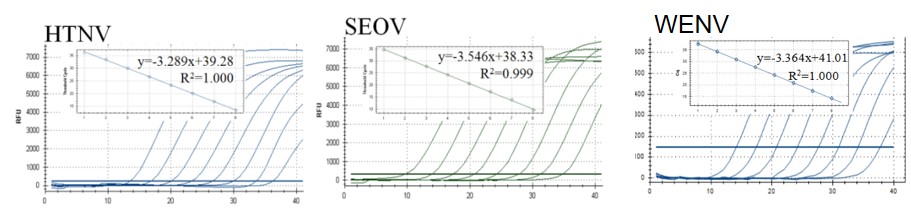


**Appendix Figure 2.** Amplification plots and standard curves of one-step real-time TaqMan RT-PCR assays. The one-step realtime TaqMan RT-PCR assays were tested using synthesized in vitro target viral RNA transcripts ranging from 10^1^ to 10^8^ copies/mL. A PCR baseline subtractive curve fit view of the data is shown with relative fluorescence units (RFUs) plotted against cycle numbers. Standard curves generated from the Ct values obtained against known concentrations, the coefficient of determination (R^2^) and slope of the regression curve for each assay are indicated.
